# Supplementary material for: Biomarkers of mortality in adults and adolescents with advanced HIV in sub-Saharan Africa
Source: Nat Commun. 2024 Jun 28;15:5492. doi: 10.1038/s41467-024-49317-7 (PMC11214617; doi:10.1038/s41467-024-49317-7)
Supplement: Supplementary file 1 — Supplementary Information [file 41467_2024_49317_MOESM1_ESM.pdf]

SUPPLEMENTARY MATERIAL

Supplementary Table 1: Time to death by cause

Cause	N	Days until death - median (IQR)
All cause	169	41 (20, 68)
TB	61	32 (19, 64)
Cryptococcosis	14	45 (23, 68)
Severe bacterial infection	21	20 (14, 50)
Other	53	45 (23, 74)
Unknown	70	46 (24, 80)

Supplementary Table 2: Best subsets regression models

Cause	Model from stepwise selection	Best model	Difference in AIC	Ranking by AIC (1=lowest AIC)	Total possible models
All cause	CRP, IFNG, IL2, IL6, IL23, IP10, Rantes	IP10, IL6, IL18, IL2, Rantes, IL23, CRP	1.7	4	5,379,616
TB	CRP, ST2	CRP, ST2	-	1	595
Cryptococcosis	IL4, IL8	IL4, IL8	-	1	595
Severe bacterial infection	CRP, CD163	CRP, CD163	-	1	595
Other	IL9, IFNG, CD14	IL9, IL18, CD14	3.4	3	6,545
Unknown	TNFA, IL18, Rantes, CD14, IFABP	IL7, IL8, Rantes, CD14, IFABP	5.3	31	277,984

Supplementary Figure 1: CONSORT diagram

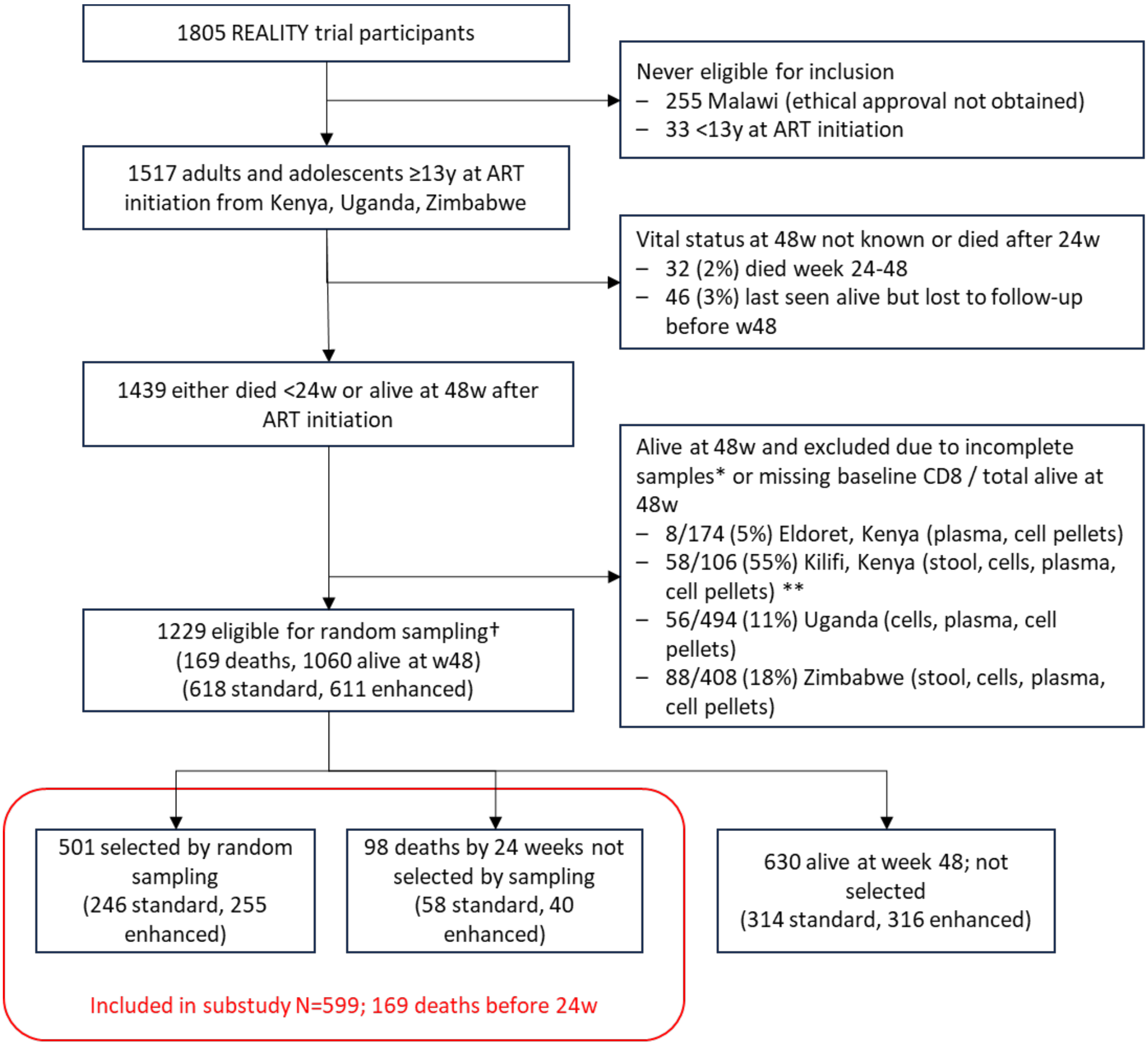

* incomplete samples defined as missing any of baseline, week 4 or week 12 for the sets of samples stated in those known to be alive at w48.

** baseline CD8 missing in 30 due to reagent unavailability; excluded on the basis of missing samples only 28/106 (26%)

† Random sampling (stratified by site and CD4 count (0-24, 25-49, 50-99 cells/mm^3; approximate terciles)) comprised 45% of participants from sites storing stool, buffy coat cells, plasma and baseline cell pellet (90% from Kilifi, Kenya because of missing baseline $CD8^+$ due to reagent unavailability), 45% from sites storing buffy coat cells and plasma/cell pellets (but not stool) (Uganda), and 10% from one single site storing plasma/cell pellets only (Eldoret, Kenya).

Supplementary Figure 2: Spearman correlation of biomarkers

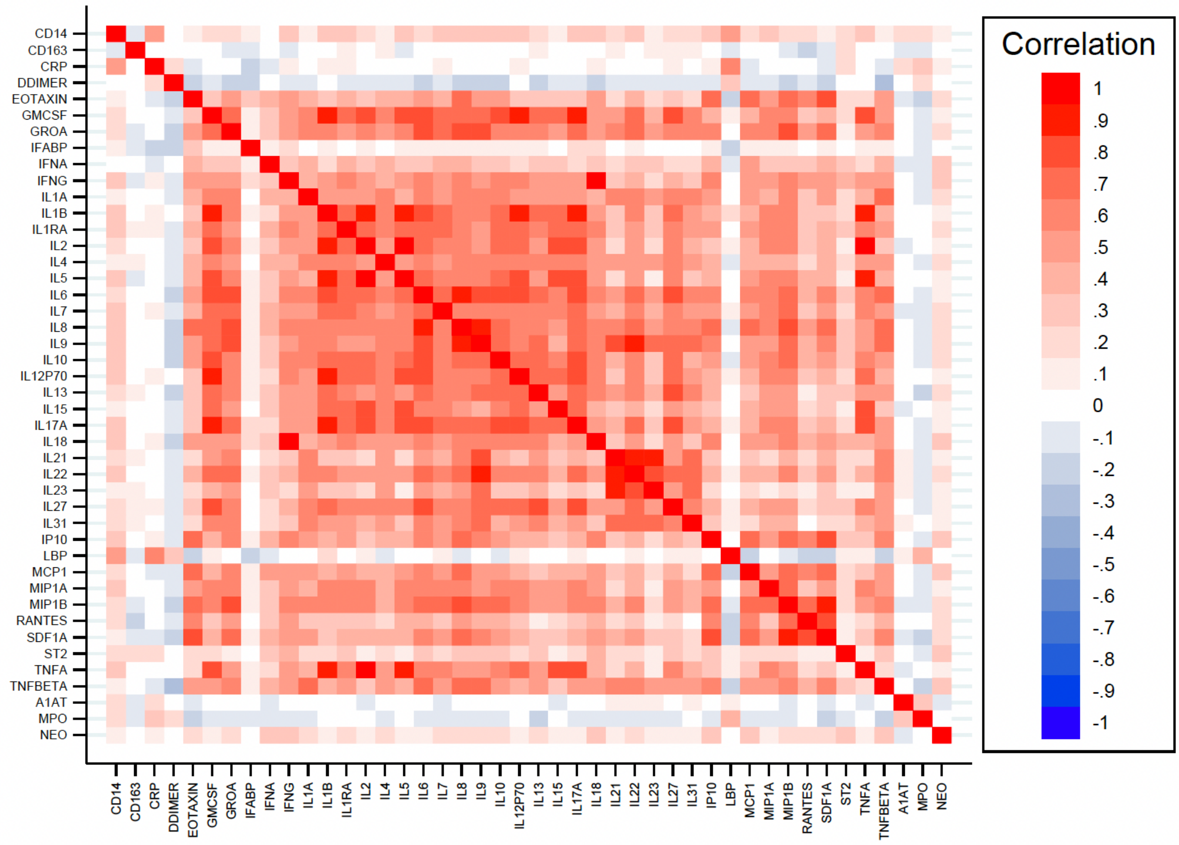

Supplementary Figure 3: Principal components scree plot

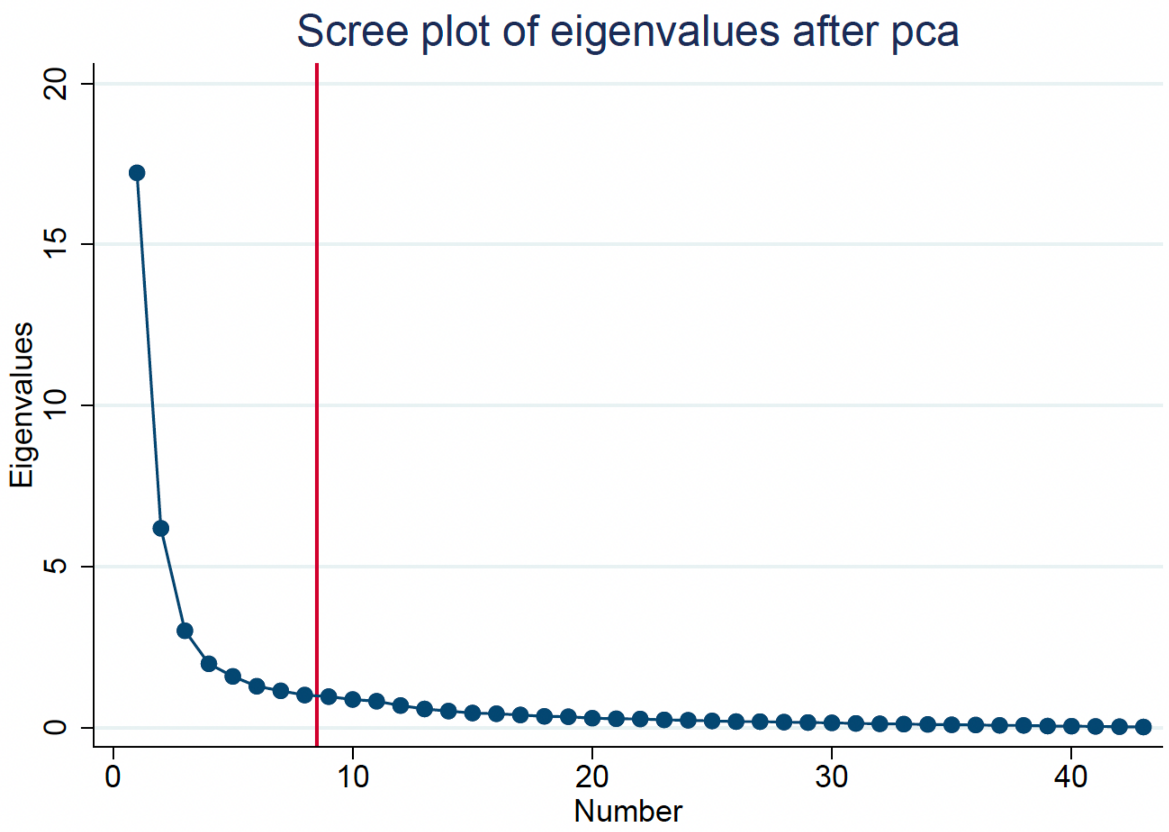

The red line shows the cutoff of the first 8 principal components selected to use for analysis.

Supplementary Figure 4: Principal components factor loadings

	Comp1	Comp2	Comp3	Comp4	Comp5	Comp6	Comp7	Comp8
CD4	0.01	-0.05	-0.12	0.15	-0.26	0.32	0.31	0.03
VL	0.03	0.04	0.14	0.09	0.19	-0.16	-0.38	0.39
IL4	0.20	-0.07	-0.03	-0.02	0.14	0.07	-0.10	-0.14
IL12P70	0.20	-0.08	-0.01	-0.09	0.02	0.10	-0.14	-0.21
IL10	0.20	-0.03	0.02	-0.08	0.06	-0.04	0.00	-0.01
IP10	0.08	0.33	0.04	0.09	0.03	-0.06	0.12	0.08
MCP1	0.09	0.34	-0.00	-0.06	-0.01	0.08	-0.07	-0.02
MIP1A	0.13	0.26	0.04	-0.08	-0.09	0.12	-0.07	-0.16
IL27	0.20	-0.14	-0.06	0.04	0.03	0.05	-0.06	-0.10
IL31	0.18	0.05	-0.08	0.22	-0.09	0.03	-0.08	-0.00
IL13	0.20	-0.08	-0.05	0.01	0.08	0.13	-0.18	-0.27
IL5	0.19	-0.06	0.07	-0.31	-0.17	0.01	0.13	0.17
GMCSF	0.21	-0.12	-0.03	-0.05	-0.04	0.07	-0.03	-0.04
IL7	0.15	0.22	0.02	-0.04	-0.15	0.01	-0.10	0.08
IL8	0.18	0.21	-0.04	0.09	0.02	-0.00	0.12	-0.04
GROA	0.19	0.11	-0.12	0.13	-0.13	-0.04	0.14	0.03
IFNA	0.12	0.06	-0.04	-0.10	0.15	-0.07	-0.48	0.24
IFNG	0.17	-0.01	0.16	-0.07	0.27	-0.16	0.07	-0.29
EOTAXIN	0.09	0.30	-0.01	-0.02	0.18	-0.04	0.19	-0.01
IL9	0.20	-0.11	-0.12	0.16	0.02	-0.10	0.14	0.10
IL6	0.22	-0.03	0.02	0.04	0.04	0.05	0.07	-0.09
IL15	0.17	-0.02	-0.03	-0.17	-0.13	-0.00	-0.10	0.22
IL1RA	0.17	0.03	0.18	-0.06	0.04	-0.04	0.03	0.12
IL21	0.16	-0.16	-0.15	0.31	-0.01	-0.14	0.05	0.12
IL18	0.17	-0.03	0.14	-0.05	0.29	-0.15	0.03	-0.32
SDF1A	0.09	0.35	-0.08	0.02	-0.08	0.05	-0.04	-0.04
IL1A	0.18	0.01	-0.07	0.11	-0.00	0.01	-0.20	0.02
IL1B	0.19	-0.18	0.05	-0.15	-0.05	0.01	0.07	0.07
TNFBETA	0.17	-0.01	-0.14	0.16	0.10	-0.09	0.02	0.04
TNFA	0.19	-0.11	0.10	-0.30	-0.14	0.04	0.09	0.09
IL2	0.18	-0.10	0.06	-0.30	-0.16	0.08	0.09	0.14
RANTES	0.07	0.28	-0.08	-0.02	0.01	-0.10	0.20	0.21
MIP1B	0.11	0.33	-0.04	-0.00	-0.11	0.10	-0.08	-0.12
IL17A	0.21	-0.09	0.01	-0.10	-0.06	0.08	0.06	0.06
IL22	0.20	-0.13	-0.11	0.19	-0.02	-0.07	0.02	0.05
IL23	0.15	-0.13	-0.18	0.35	-0.01	-0.05	0.03	0.11
CD14	0.07	0.00	0.34	0.08	-0.14	-0.40	0.13	0.11
CRP	0.04	-0.00	0.43	0.21	-0.19	-0.04	0.04	-0.18
LBP	0.04	0.00	0.43	0.19	-0.26	-0.09	-0.06	-0.03
ST2	0.07	0.01	0.30	0.11	0.38	0.13	0.23	0.09
DDIMER	-0.00	-0.01	0.31	0.12	0.00	0.27	-0.12	0.20
CD163	0.03	-0.01	0.12	0.06	0.39	0.52	0.16	0.27
IFABP	-0.02	0.02	-0.16	-0.23	0.22	-0.35	0.24	0.09

Loadings
.7
.6
.5
.4
.3
.2
.1
0
-.1
-.2
-.3
-.4
-.5
-.6
-.7

REALITY TRIAL TEAM

The REALITY Trial Team who were not authors on the current manuscript are:

Joint Clinical Research Centre (JCRC), Kampala, Uganda (coordinating centre for Uganda): P Mugyenyi, C Kityo, P Wavamunno, E Nambi, P Ocitti, M Ndigendawani.

JCRC, Fort Portal, Uganda: S Kabahenda, M Kemigisa, J Acen, DOlebo, G Mpamize, A Amone, D Okweny, A Mbonye, F Nambaziira, A Rweyora, M Kangah and V Kabaswahili.

JCRC, Gulu, Uganda: J Abach, G Abongomera, J Omongin, I Aciro, A Philliam, B Arach, E Ocung, G Amone, P Miles, C Adong, C Tumsuiime, P Kidega, B Otto, F Apio.

JCRC, Mbale, Uganda: K Baleeta, A Mukuye, M Abwola, F Ssennono, D Baliruno, S Tuhirwe, R Namisi, F Kigongo, D Kikyonkyo, F Mushahara, D Okweny, J Tusiime, A Musiime, A Nankya, D Atwongyeire, S Sirikye, S Mula, N Noowe.

JCRC, Mbarara, Uganda: A Lugemwa, M Kasozi, S Mwebe, L Atwine, T Senkindu, T Natuhurira, C Katemba, E Ninsiima, M Acaku J Kyomuhangi, R Ankunda, D Tukwasibwe, L Ayesiga.

University of Zimbabwe Clinical Research Centre, Harare, Zimbabwe: J Hakim, K Nathoo, A Reid, E Chidziva, T Mhute, GC Tinago, J Bhiri, S Mudzingwa, M Phiri, J Steamer, R Nhema, C Warambwa, G Musoro, S Mutsai, B Nemasango, C Moyo, S Chitongo, K Rashirai, S Vhembo, B Mlambo, S Nkomani, B Ndemera, M Willard, C Berejena, Y Musodza, P Matiza, B Mudenge, V Guti.

KEMRI Wellcome Trust Research Programme, Kilifi, Kenya: A Etyang, C Agutu, K Maitland, P Njuguna, S Mwaringa, T Etyang, K Awuondo, S Wale, J Shangala, J Kithunga, S Mwarumba, S Said Maitha, R Mutai, M Lozi Lewa, G Mwambingu, A Mwanzu, C Kalama, H Latham, J Shikuku, A Fondo, A Njogu, C Khadenge, B Mwakisha.

Moi University Clinical Research Centre, Eldoret, Kenya: K Wools-Kaloustian, W Nyandiko, P Cheruiyot, A Sudoi, S Wachira, B Meli, M Karoney, A Nzioka, M Tanui, M Mokaya, W Ekiru, C Mboya, D Mwimali, C Mengich, J Choge, W Injera, K Njenga, S Cherutich, M Anyango Orido,G Omondi Lwande, P Rutto, A Mudogo, I Kutto, A Shali, L Jaika, H Jerotich, M Pierre.

Department of Medicine and Malawi-Liverpool- Wellcome Trust Clinical Research Programme, College of Medicine, Blantyre, Malawi: J Mallewa, S Kaunda, J Van Oosterhout, B O'Hare, R Heydermann, C Gonzalez, N Dzabala, C Kelly, B Denis, G Selemani, L Nyondo Mipando, E Chirwa, P Banda, L Mvula, H Msuku, M Ziwoya, Y Manda, S Nicholas, C Masesa, T Mwalukomo, L Makhaza, I Sheha, J Bwanali, M Limbuni.

Trial Coordination and Oversight: MRC Clinical Trials Unit at UCL, London, UK: M Thomason, S Pett, A Griffiths, H Wilkes, C Rajapakse, M Spyer, Data Management Systems: M Rauchenberger, N Van Looy, E Little, K Fairbrother.

Social Science Group: F Cowan, J Seeley, S Bernays, R Kawuma, Z Mupambireyi.

Independent REALITY Trial Monitors: F Kyomuhendo, S Nakalanzi, J Peshu, S Ndaa, JChabuka, N Mkandawire, L Matandika, C Kapuya**.**

Trial Steering Committee: I Weller (Chair), E Malianga, C Mwansambo, F Miiro, P Elyanu, E Bukusi, E Katabira, O Mugurungi, J Hakim, A Etyang, P Mugyenyi, J Mallewa.

Data Monitoring Committee: T Peto (Chair), P Musoke, J Matenga, S Phiri.

Endpoint Review Committee (independent members): H Lyall (Co-Chair), V Johnston (Co-Chair), F Fitzgerald, F Post, F Ssali, A Arenas-Pinto, A Turkova, A Bamford.
