# Supplementary material for: Biomarkers of mortality in adults and adolescents with advanced HIV in sub-Saharan Africa
Source: Nat Commun. 2024 Jun 28;15:5492. doi: 10.1038/s41467-024-49317-7 (PMC11214617; doi:10.1038/s41467-024-49317-7)
Supplement: Supplementary file 3 — Description of Additional Supplementary Files [file 41467_2024_49317_MOESM3_ESM.docx]

File name: Supplementary Data 1

Description: Stata files for all analyses conducted in this manuscript

File name: Supplementary Data 2

Description: Data used to produce all Figures in this manuscript
