# Supplementary material for: Biomarkers of mortality in adults and adolescents with advanced HIV in sub-Saharan Africa
Source: Nat Commun. 2024 Jun 28;15:5492. doi: 10.1038/s41467-024-49317-7 (PMC11214617; doi:10.1038/s41467-024-49317-7)
Supplement: Supplementary file 6 — Reporting Summary [file 41467_2024_49317_MOESM6_ESM.pdf]

nature portfolio

Corresponding author(s): Andrew J Prendergast

Last updated by author(s): 27 April 2024

Reporting Summary

Nature Portfolio wishes to improve the reproducibility of the work that we publish. This form provides structure for consistency and transparency in reporting. For further information on Nature Portfolio policies, see our Editorial Policies and the Editorial Policy Checklist.

Statistics

For all statistical analyses, confirm that the following items are present in the figure legend, table legend, main text, or Methods section.

n/a	Confirmed	
☐	☒	The exact sample size (*n*) for each experimental group/condition, given as a discrete number and unit of measurement
☐	☒	A statement on whether measurements were taken from distinct samples or whether the same sample was measured repeatedly
☐	☒	The statistical test(s) used AND whether they are one- or two-sided<br>*Only common tests should be described solely by name; describe more complex techniques in the Methods section.*
☐	☒	A description of all covariates tested
☐	☒	A description of any assumptions or corrections, such as tests of normality and adjustment for multiple comparisons
☐	☒	A full description of the statistical parameters including central tendency (e.g. means) or other basic estimates (e.g. regression coefficient) AND variation (e.g. standard deviation) or associated estimates of uncertainty (e.g. confidence intervals)
☐	☒	For null hypothesis testing, the test statistic (e.g. *F*, *t*, *r*) with confidence intervals, effect sizes, degrees of freedom and *P* value noted<br>*Give P values as exact values whenever suitable.*
☒	☐	For Bayesian analysis, information on the choice of priors and Markov chain Monte Carlo settings
☒	☐	For hierarchical and complex designs, identification of the appropriate level for tests and full reporting of outcomes
☒	☐	Estimates of effect sizes (e.g. Cohen's *d*, Pearson's *r*), indicating how they were calculated

Our web collection on statistics for biologists contains articles on many of the points above.

Software and code

Policy information about availability of computer code

Data collection	Laboratory data were compiled and cleaned in MS Excel prior to analysis
Data analysis	Stata version 16.1, and R version 4.2.2

For manuscripts utilizing custom algorithms or software that are central to the research but not yet described in published literature, software must be made available to editors and reviewers. We strongly encourage code deposition in a community repository (e.g. GitHub). See the Nature Portfolio guidelines for submitting code & software for further information.

Data

Policy information about availability of data

All manuscripts must include a data availability statement. This statement should provide the following information, where applicable:

- Accession codes, unique identifiers, or web links for publicly available datasets
- A description of any restrictions on data availability
- For clinical datasets or third party data, please ensure that the statement adheres to our policy

Source data are provided with this paper. The full dataset used in the analyses presented in this manuscript are available on Figshare https://doi.org/10.6084/m9.figshare.24459760

Research involving human participants, their data, or biological material

Policy information about studies with human participants or human data. See also policy information about sex, gender (identity/presentation), and sexual orientation and race, ethnicity and racism.

Reporting on sex and gender	Participants of any sex or gender were eligible for inclusion in the REALITY trial, from which specimens and data for the current study are derived; and the study recruited roughly equal numbers of self-identified men and women (51% vs 49%). Since this is an exploratory analysis, which is a sub-study of a randomized trial, we did not pre-specify an analysis stratified by sex or gender.
Reporting on race, ethnicity, or other socially relevant groupings	The study was conducted in 4 African countries, therefore the participants are primarily black Africans. The participants were recruited from a number of different sites in each of the countries but without consideration of race or ethnicity or focusing on any key populations.
Population characteristics	The REALITY trial in which this immunology sub-study is embedded recruited ART-naïve HIV-infected adults and children who were 5 years or older, with a CD4 count <100 cells per mm3. Participants were randomized at ART initiation to three interventions in a 2x2x2 factorial design: enhanced antimicrobial prophylaxis, adjunctive raltegravir therapy, and ready-to-use supplementary food. Participants were recruited from countries in sub Saharan Africa that have a high ongoing prevalence of HIV infection.
Recruitment	Recruitment into the main REALITY trial has been previously reported (Hakim J et al., N Engl J Med 2017). For this sub-study, we selected all 169 deaths occurring by 24 weeks in the study centres in Kenya, Uganda and Zimbabwe (ethical approval could not be obtained for the substudy in Malawi as the trial had closed) as cases, and a random sample of non-cases who remained in follow-up (i.e., alive) until 48 weeks with complete sets of samples to week 24 and data on baseline CD8+ T-cell counts.
Ethics oversight	The trial and the laboratory work in this study were approved by ethics committees in Kenya (Moi University Institutional Research and Ethics Committee and the Kenya Medical Research Institute Ethics Review Committee), Uganda (Joint Clinical Research Centre Institutional Review Board and the Uganda National Council for Science and Technology), Zimbabwe (Joint Parirenyatwa Hospital and College of Health Sciences Research Ethics Committee and the Medical Research Council of Zimbabwe), and the UK (University College London Ethics Committee). Adult participants and parents/guardians provided written informed consent to enrol in the trial, which included storage of biological specimens for subsequent analysis; older children provided additional assent, according to national guidelines.

Note that full information on the approval of the study protocol must also be provided in the manuscript.

Field-specific reporting

Please select the one below that is the best fit for your research. If you are not sure, read the appropriate sections before making your selection.

☒ Life sciences ☐ Behavioural & social sciences ☐ Ecological, evolutionary & environmental sciences

For a reference copy of the document with all sections, see nature.com/documents/nr-reporting-summary-flat.pdf

Life sciences study design

All studies must disclose on these points even when the disclosure is negative.

Sample size	The target sample size for plasma baseline biomarkers was 602 patients (actual 599 due to natural variability in the random sampling). This provided 80% power to detect a hazard ratio for mortality associated with each biomarker quartile of 0.78, adjusting for the case-cohort design
Data exclusions	Participants without complete sets of samples to week 24 and data on baseline CD8+ T-cell counts were excluded
Replication	All biomarker measurements were done according to the kit manufacturer's instractions and appropriate assay quality controls used as highlighted in the methods. Any deviations were included in the methods. Any ssay results not meeting QC criteria were repeated to ensure they met the assay criteria for inclusion. Assay controls were replicated and reproducible within the acceptible range specified by the kit manufacturer.
Randomization	The case-cohort design aimed first to randomly sample 45% of participants from sites storing stool, buffy coat cells, plasma and baseline cell pellet (90% from one of these two sites because of missing CD8+ due to reagent unavailability), 45% from sites storing buffy coat cells and plasma/cell pellets, and 10% from one single site storing plasma/cell pellets only. Sampling was stratified by CD4 count (0-24, 25-49, 50-99 cells/mm3; approximate terciles). Any deaths by 24 weeks not selected by the random sampling were added to the sample (total N=599).
Blinding	The main REALITY trial has been previously reported and was not blinded due to the nature of the interventions being provided. For the current substudy, all available baseline and 4 weeks post-ART initiation samples were retrieved and assayed by laboratory scientists who were blinded to trial arm and clinical outcomes

Reporting for specific materials, systems and methods

We require information from authors about some types of materials, experimental systems and methods used in many studies. Here, indicate whether each material, system or method listed is relevant to your study. If you are not sure if a list item applies to your research, read the appropriate section before selecting a response.

Materials & experimental systems

n/a	Involved in the study	
☒	☐	Antibodies
☒	☐	Eukaryotic cell lines
☒	☐	Palaeontology and archaeology
☒	☐	Animals and other organisms
☐	☒	Clinical data
☒	☐	Dual use research of concern
☒	☐	Plants

Methods

n/a	Involved in the study	
☒	☐	ChIP-seq
☒	☐	Flow cytometry
☒	☐	MRI-based neuroimaging

Clinical data

Policy information about clinical studies

All manuscripts should comply with the ICMJEguidelines for publication of clinical research and a completedCONSORT checklist must be included with all submissions.

Clinical trial registration	The REALITY Trial (ISRCTN43622374)
Study protocol	The REALITY Trial protocol can be accessed here: https://www.mrcctu.ucl.ac.uk/media/1293/reality-protocol.pdf
Data collection	The trial was undertaken between 2013 and 2016 in Kenya, Malawi, Uganda and Zimbabwe and recruited ART-naïve HIV-infected adults and children who were 5 years or older with a CD4 count <100 cells per mm3.
Outcomes	The intervention bundle conferred a 27% relative reduction in mortality by 24 weeks (primary trial outcome), and 24% mortality reduction by 48 weeks.
